# Diversity and compositional differences of the airborne microbiome in a biophilic indoor environment

**DOI:** 10.1038/s41598-023-34928-9

**Published:** 2023-05-20

**Authors:** Akinobu Toyoda, Yusuke Shibata, Yuzy Matsuo, Kumi Terada, Hiroki Sugimoto, Koichi Higashi, Hiroshi Mori, Akinori Ikeuchi, Masakazu Ito, Ken Kurokawa, Satoshi Katahira

**Affiliations:** 1 Frontier Research Center, Toyota Motor Corporation, Toyota, Aichi 471-8572 Japan; 2 Toyota Central R&D Labs, Inc., Nagakute, Aichi 480-1192 Japan; 3 Department of Informatics, National Institute of Genetics, Mishima, Shizuoka 411-8540 Japan

**Keywords:** Microbiome, Air microbiology

## Abstract

Biophilic design based on indoor planting plays an important role in human physical and mental well-being. To investigate and assess the effects of indoor planting on air quality, we sequenced 16S rRNA gene amplicons to compare the airborne bacterial microbiomes of three planting rooms before and after installing natural materials (plants, soil, water, etc.) with distinct biophilic attributes. Incorporation of indoor plantings significantly increased the taxonomic diversity of the airborne microbiome in each room, and we observed different microbiome compositions in each room. The proportional contribution of each bacterial source to the airborne microbiome in the indoor planting rooms was estimated by SourceTracker2. This analysis revealed that the proportion of airborne microbial sources (e.g., plants and soil) varied depending on the natural materials installed. Our results have important implications for indoor planting with biophilic design to control the indoor airborne microbiome.

## Introduction

Humans spend about 90% of their lifetimes in indoor spaces^1^. Humans are influenced by their indoor environments such as design and air quality, and numerous studies have shown a relationship between indoor environment and human performance^2,3^. Biophilia hypothesis, that suggests that humans instinctively look for a connection with nature, has attracted attention^4^. Biophilic design based on this hypothesis is a method of incorporating elements of nature into living and/or working spaces and has attracted attention because this may strengthen an individual’s connection with nature and promote a healthier life both physically and mentally. For example, implementation of green design and application of nature-derived olfactory stimulants have been reported to reduce stress and increase worker productivity^5–8^. Moreover, there is a study on the mental fatigue reduction in indoor planting rooms^9^.

Bioaerosols, including the airborne microbiome, are considered factors of the indoor environment that influence humans^10^. Recent advances in bioaerosol sampling and DNA sequencing technology have made it possible to analyze the airborne microbiome at low concentrations^11–13^. Use of these techniques has revealed that the phylogenetic diversity of the indoor-air microbiome is lower than that of the outdoor air and that human-related microbes predominate^14,15^. Because humans have largely shifted their living spaces from rural areas to urban areas and now spend most of their lives in indoor spaces, humans are exposed to bioaerosols specific to indoor environment^16^. Bioaerosols may affect human health for better or worse, and the diversity of the airborne microbiome has been emphasized as an especially important factor^17–19^. A typical example of this influence is the hygiene hypothesis, which proposes that childhood exposure to environments that are too clean is involved in the development of allergic diseases^20^. It has also been suggested that exposure to a variety of microbes may reduce the incidence of atopy and asthma^21,22^. Thus, control of the indoor airborne microbiome is a prerequisite factor for creating a human-friendly environment^23^.

Biophilic design using real plants can be applied as a means to alter the microbiome of an indoor environment. Numerous microbes are attached to plant leaves and soils, and some studies have shown that airborne microbiomes in indoor environments are changed by plants^24,25^. However, the effects of various natural materials (plants, soil, water, etc.) installed in indoor environments on airborne microbiomes have not been thoroughly investigated. In previous study, we designed and constructed three types of experimental indoor rooms, which were introduced to plants with roughly similar leaf surface and shape^9,26^. In this study, we conducted an experiment aimed at investigating the effects of different natural materials on the airborne microbiome in these biophilic indoor environments. The airborne bacterial microbiome in each experimental room before and after planting was analyzed using 16S rRNA gene amplicon sequencing. The stability and sources of the airborne microbiomes in the experimental rooms were also investigated.

## Results

**Figure 1:**
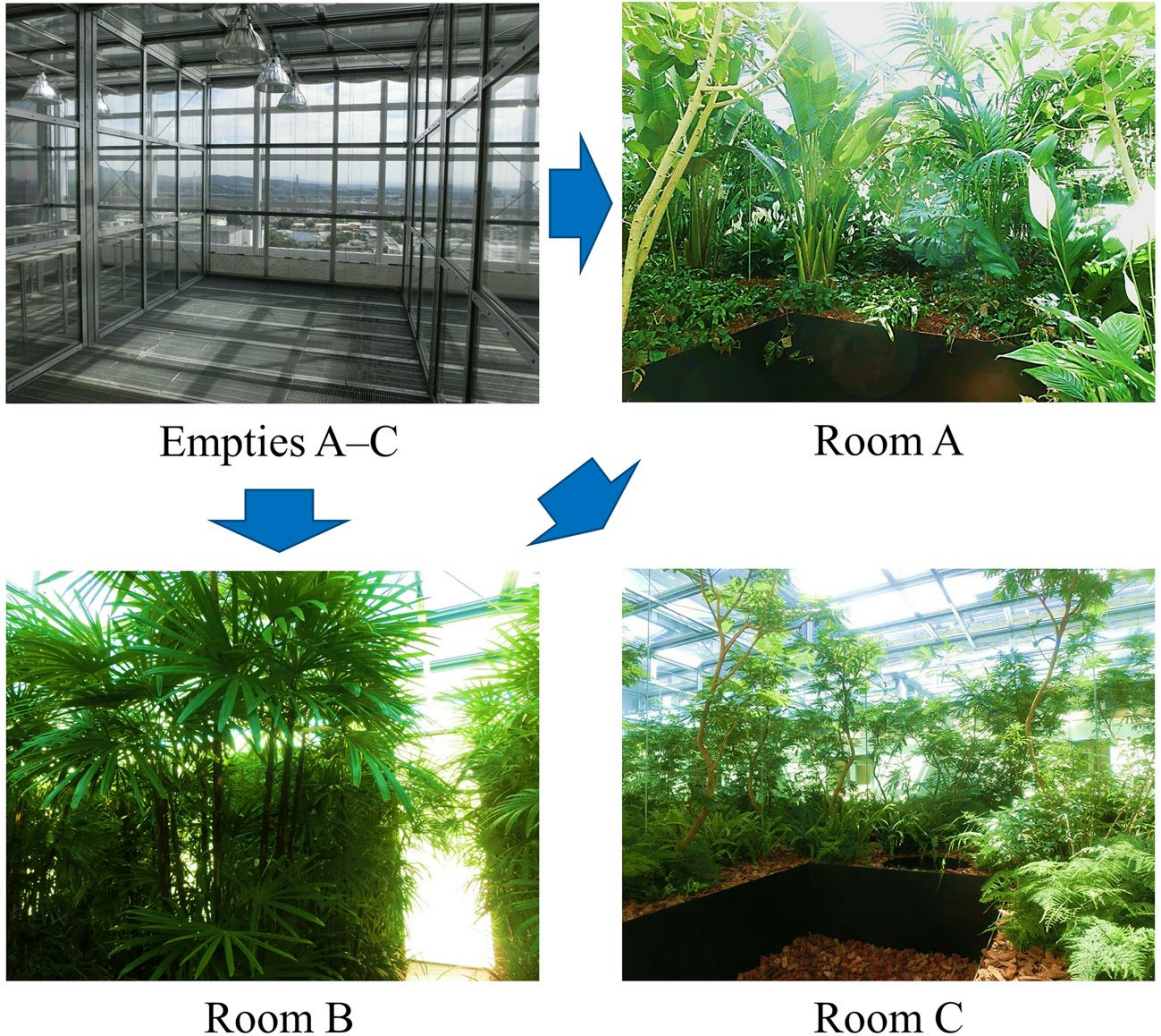
Planting rooms design. The representative image of empty room (Empties A–C) was taken in Empty B in February, and the representative images of Rooms (Rooms A–C) were taken in March. Each planting room was composed of different kinds of natural materials and layouts. See also Supplementary Fig. S1 and Supplementary Table S1**.**

In this study, a total of 78 air samples were collected from the three empty and planting rooms (Fig. 1, Supplementary Fig. S1, and Supplementary Table S1) during the measurement period. Sequencing of the 16S rRNA gene amplicon in air samples yielded 5,422,387 reads (Supplementary Fig. S2a). Rarefaction curves showed that these reads were sufficient to infer the structure and relative abundance of the microbiota in each air sample (Supplementary Fig. S2b).

### Diversity and microbial composition of airborne microbiomes

**Figure 2:**
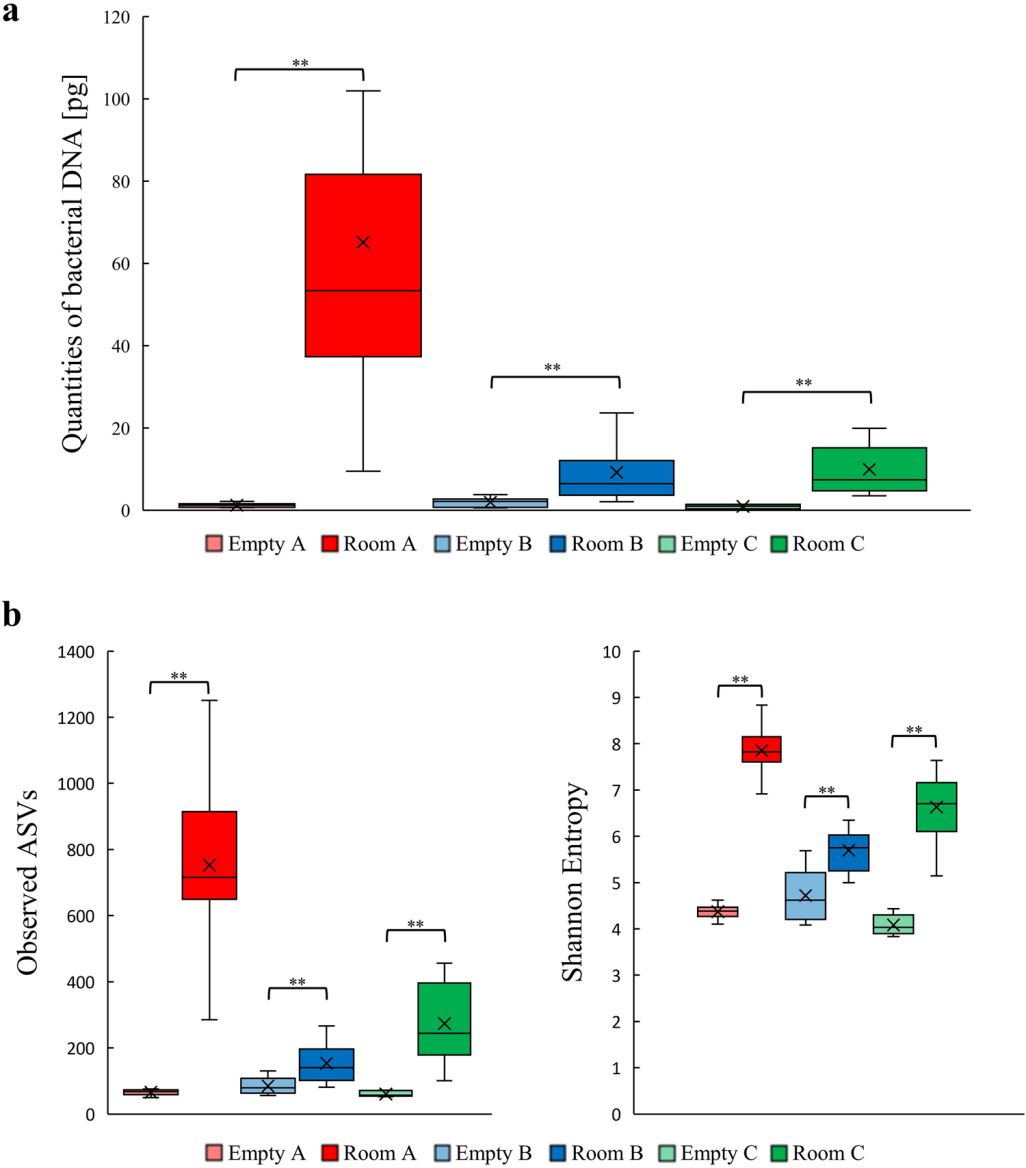
Comparison of the microbiota abundance and diversity in airborne samples of each room. (**a**) Comparison of DNA quantities based on qPCR analysis and (**b**) observed ASVs and Shannon entropy in the airborne microbiomes of Empties A–C and Rooms A–C. The box plots indicate the median and interquartile range, and the whiskers extend to the most extreme data point within 1.5 × of the inter quartile range of the first (lower whisker) or third (upper whisker) quartile. Statistical analysis was performed using Kruskal–Wallis pairwise test. Statistical significance between empty and planting rooms is denoted with ** (*P* < 0.01).

The quantities of bacterial DNA differed significantly between the empty and planting rooms (Fig. 2a, Kruskal–Wallis pairwise test; *P* = 0.0003 (Empty A vs. Room A); *P* = 0.0020 (Empty B vs. Room B); *P* = 0.0003 (Empty C vs. Room C)). Similarly, the total number of observed amplicon sequence variants (ASVs) differed significantly between the empty and planting rooms (Fig. 2b, Kruskal–Wallis pairwise test; *P* = 0.0003 (Empty A vs. Room A); *P* = 0.0035 (Empty B vs. Room B); *P* = 0.0003 (Empty C vs. Room C)). Room A had the greatest number of ASVs, followed by Room C and Room B, with the empty room having the fewest ASVs. These results indicated that natural materials in rooms increase the bacterial diversity in indoor air.

**Figure 3:**
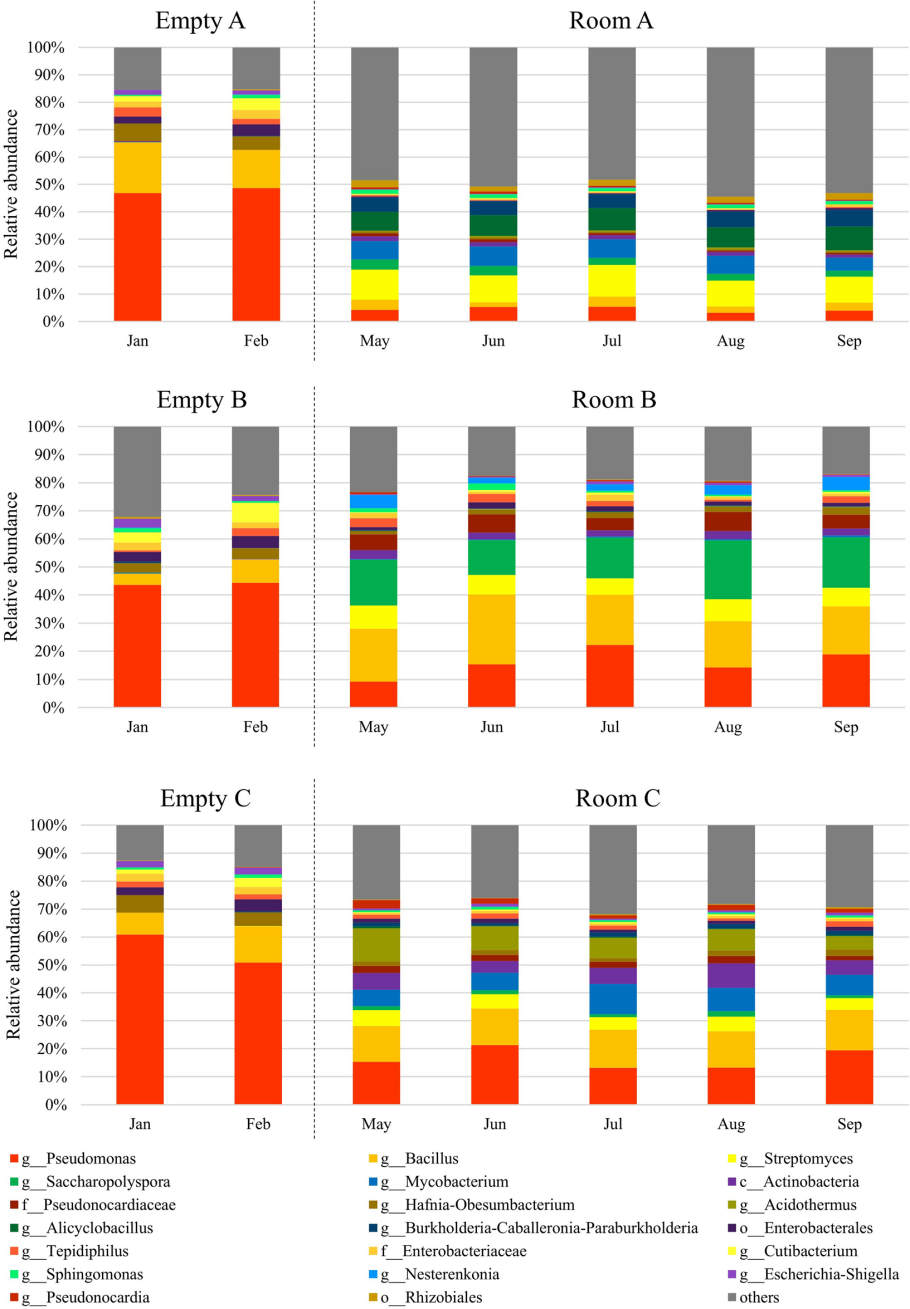
Time-series abundance of bacterial taxa in each room before and after planting. Bar plots show abundant bacterial taxa averaged for each month in empty rooms (Jan, Feb) and planting rooms (May, Jun, Jul, Aug, Sep) at the genus level or higher taxonomic level.

Next, we investigated the monthly averages of the taxonomic abundances of the airborne microbiomes in the empty and planting rooms. The microbial composition and diversity in each room changed between before and after planting. After the change, they remained stable for 5 months (Fig. 3 and Supplementary Table S2). *Pseudomonas* was the most abundant taxon in the empty rooms (~ 49%), which decreased after planting (to ~ 4%, ~ 16%, ~ 17% in Rooms A, B, and C, respectively). The characteristic taxa observed after planting were *Streptomyces* (~ 10%) and *Alicyclobacillus* (~ 8%) in Room A, *Saccharopolyspora* (~ 16%) in Room B, and *Acidothermus* (~ 8%) in Room C. Thus, indoor planting has changed and diversified the airborne microbiome at ASV level (Supplementary Table S2).

**Figure 4:**
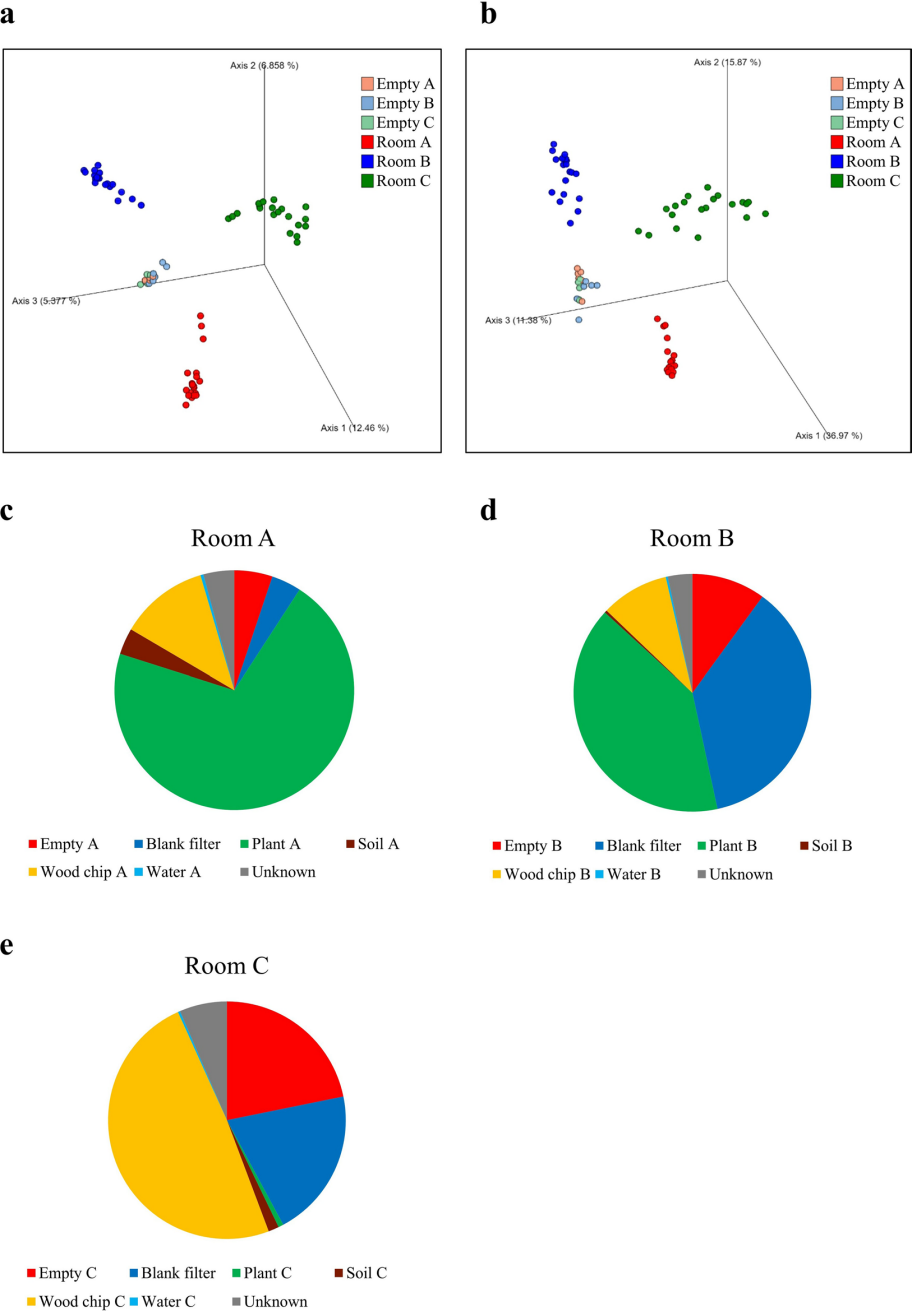
Principal coordinate analysis (PCoA) plots of taxonomic compositions of planting rooms and source-tracker analysis between empty and planting rooms. Each room was compared by PCoA using (**a**) the Jaccard distance and (**b**) Bray–Curtis dissimilarity for all samples. Plots show the coordinates of all samples in three dimensions, and the colors denote empty rooms (Empties A–C) and planting rooms (Rooms A–C) as indicated. See also Supplementary Table S3 regarding statistical analysis. (**c**–**e**) Pie charts show the proportion of sources in the airborne microbiome of each planting room by SourceTracker2.

Principal coordinate analysis (PCoA) was conducted for all measured samples using Jaccard distance and Bray–Curtis dissimilarity to confirm differences in microbial composition (Fig. 4a,b). The PCoA plots and permutational multivariate analysis of variance (PERMANOVA) showed significantly different taxonomic compositions of microbiomes among Rooms A–C in addition to differences between before and after planting (Supplementary Table S3). In particular, these plots and pseudo-F values revealed that the taxonomic composition in Room A was less variation than that in the other two planting rooms. These results indicated that indoor planting changed the composition of the airborne microbiome, and the composition depends on the natural materials installed indoors.

### Sources tracking of the airborne microbiome in the indoor planting rooms

Indoor planting changed drastically the composition of the airborne microbiome. It is therefore reasonable to predict that the composition of airborne microbiome in our experimental rooms depended on the natural materials installed indoors. We identified the proportional contribution of each bacterial source to the airborne microbiome in the indoor planting rooms by using SourceTracker2^27^, which can estimate the proportion of source microbiomes contributing to the sink microbiome based on the Bayesian framework (Fig. 4c–e). In this study, the microbiomes of the air before planting (empty), plants (leaf surfaces), soil, wood chips, water, and blank filters were used as sources (Supplementary Fig. S3) and the airborne microbiome of each planting room was used as the sink. The results showed that the airborne microbiome in Room A had the highest contribution from the microbiome of plants (“Plant A”, ~ 71%), followed by wood chips (“Wood chip A”, ~ 12%) and air before planting (“Empty A”, ~ 5%). Similarly, the airborne microbiome in Room B had the highest contribution from the microbiome of plants (“Plant B”, ~ 40%), followed by the blank filter (“Blank filter”, ~ 37%) and air before planting (“Empty B”, ~ 10%). The airborne microbiome in Room C had the highest contribution from the microbiome of wood chips (“Wood chip C”, ~ 49%), followed by air before planting (“Empty C”, ~ 22%) and the blank filter (“Blank filter”, ~ 20%). Furthermore, we found that the characteristic bacteria in air of each room were derived from each of the main sources. For example, *Streptomyces* in Room A was observed from plants, *Saccharopolyspora* in Room B was observed from plants, and *Acidothermus* in Room C was observed from wood chips. These results indicated that plants and wood chips can change the composition of the airborne microbiome in the room.

## Discussion

To investigate the effect of indoor planting based on biophilic design concept on the indoor airborne microbiome, we sampled and analyzed the microbiomes of air and the natural materials in three experimental rooms that we designed (Fig. 1). The 16S rRNA gene amplicon sequencing analysis results showed that the airborne microbiome in each planting room became more diverse than before planting (Figs. 2, 3), and taxonomic compositions of airborne microbiomes were significantly different among Rooms A–C (Fig. 4a,b). The main sources of the airborne microbiome in the indoor planting rooms were plants and wood chips, and their proportions differed in the three experimental rooms (Fig. 4c–e).

Plants can affect the airborne microbiome in indoor rooms^25,28^. In this study, we confirmed that the composition of the airborne microbiome was altered by indoor planting in each experimental room. In addition, we found that, based on the difference in installed plant materials such as plant species, the airborne microbiome of the rooms differed from each other. These results are consistent with the report that the airborne microbiome in the greenhouse located in a botanical garden has a different diversity depending on the type of vegetation^29^. Taken together, indoor airborne microbiomes are highly affected by indoor plant microbiomes.

The relationship between particulates and airborne microbiomes has been reported extensively^30–32^, and it is likely that particulates have a significant effect on microbial community composition. In this study, it was confirmed that the number of particulates in each room was different (Supplementary Fig. S4). In particular, Room A, where the quantities of bacterial DNA in the air was high, tended to have a higher number of particulates than the other rooms. This result suggests that there is some correlation between the number of particulates and the quantities of bacterial DNA. Clarifying the relationship between particulates and microbiomes in biophilic indoor environments is a topic for future research.

The airborne microbiome has been reported to undergo seasonal changes in both outdoor and indoor spaces^33,34^. However, the compositions of airborne microbiomes in our experimental rooms were maintained for ~ 5 months (Figs. 3, 4). It is interesting to note that the airborne microbiome composition remained stable for a long period of time after planting, even though the air in each room was replaced by ventilation. In addition, in the experimental rooms, there were no effects of maintenance every other week, such as replanting dead plants and pruning leaves. This may be because the experimental rooms were not easily affected by the outside air due to the ventilation by the high efficiency particulate air filter, and the environmental conditions such as temperature and humidity were kept constant. It also suggested that the microorganisms were continually being supplied into the air from indoor sources. On the other hand, it has not been investigated whether the introduction of the same components will result in the same airborne microbiome composition each time. Reproducibility of space construction is an important issue for controlling airborne microbiome of indoor space. Moreover, it is also necessary to understand the effects of varying the amount of introduction in the same component, and these will be the next research topics.

The main sources of the airborne microbiomes in the experimental rooms, as inferred by SourceTracker2, were different in each room. In particular, the airborne microbiome in Room A was more affected by plants than was in the other rooms. This may be caused by the characteristics of plants introduced in Room A which have relatively large leaf surface areas compared to the other rooms although direct evidence for that is still lacking (Supplementary Table S4). The different types of wood chips used in each room may have also influenced the source of the airborne microbiome. Airborne microbiomes in forest environments are influenced by soil as well as plants^35^, but soil-derived microbes were hardly observed in our experimental rooms. This discrepancy was probably because the wood chips covered the soil. These results imply that the composition of the airborne microbiome might be depended on the type and arrangement of natural materials introduced into indoor spaces.

In this analysis by SourceTracker2, the blank filters were significant sources for the airborne microbiomes in Rooms B and C. In addition, the blank filters contributed ~ 80% or more to the empty rooms (Supplementary Fig. S5). This may be due to the relatively large impact of experimental contamination by the lower DNA concentration of the airborne microbiomes in Rooms B, C, and the empty rooms. Care should be taken when analyzing low biomass samples such as airborne microbiome, because low biomass samples are critically impacted by experimental contamination^36^. In particular, the quantities of bacterial DNA of the airborne microbiomes in the empty rooms (mean ± standard deviation, 1.40 ± 0.86 pg) were as low as those of the microbiomes of the blank filters (1.11 ± 0.12 pg), so it was inferred that the airborne microbiome in the empty rooms and the microbiomes of the blank filters were similar. It should also be noted that the breakdown of the total contribution rate of the blank filters and the empty rooms in this analysis may not be accurate because the above makes it difficult to distinguish between the blank filters and the empty rooms in SourceTracker2. However, although the microbes in the blank filter (e.g., *Pseudomonas* and *Bacillus*) affected the microbiome composition in all rooms (Fig. 3), the major taxa differed between rooms A, B, and C after excluding the two genera. This difference in microbiome composition indicates that the plant composition affects the air microbiome.

Contact with nature such as forest bathing^37^ is considered to have a positive effect on humans. There are also studies on the visual and olfactory effects of nature^5,6,38,39^. Since humans today spend most of their time in indoor spaces^1^, biophilic design, which incorporates elements that have the positive effects of nature into indoor spaces, has been attracting attention^40^ and is expected to improve their well-being. Furthermore, several reports have emerged showing that nature's diverse microorganisms have positive effects on people, and some studies have suggested a relationship between airborne microbiomes and human health^21,22,41,42^. Therefore, if we can control the airborne microbiome in addition to visual and olfactory stimuli in the indoor environment, we will be able to realize better biophilic design for humans. The results of this study suggest that the design of indoor planting schemes is important for controlling the airborne microbiome in indoor environments.

In conclusion, to investigate the effects of indoor planting on air quality, we sampled and analyzed the microbiome of air and natural materials in three experimental rooms that we designed. We found that the composition of the airborne microbiome and the contribution of each planting element varied depending on the natural materials installed in the experimental room. The results suggest that the airborne microbiome in indoor environments might be controlled by indoor plantings, providing insight into the establishment of diverse airborne microorganisms in indoor environments. In the future, if we can establish a methodology to control the airborne microbiome by clarifying whether it is possible to reproduce the airborne microbiome composition and what are the key factors in changing and maintaining the airborne microbiome, we will be one step closer to creating a biophilic indoor environment that benefits human well-being.

## Methods

### Building the biophilic indoor rooms

We constructed three types of rooms in the biotron greenhouse. The width, depth, and height of each room were 3 m, 5 m, and 3 m, respectively. Temperature and relative humidity in each room were controlled to be 25 °C and 55% RH, respectively. The three rooms were constructed based on different concepts^9^ with Pasona Panasonic Business Service Co., Ltd (Tokyo, Japan). The plants in each room were different in type, combination, and arrangement, and different wood chips were used in each room. In addition, water basins, channels, and waterfalls were installed in each room, and water was designed to circulate within each room (Supplementary Fig. S1 and Supplementary Table S1). Plants were purchased from parkERs Park Corporation (Tokyo, Japan) and introduced into each room in March 2019. Plant maintenance was performed every two weeks and included watering, pruning, replacing dead plants, and spraying with GM-2000 (Kaiyu, Chiba, Japan), a plant maintenance chemical.

### Sample collection

To sample the airborne microbiome, a total of 3000 L air was collected at average rate of 50 L/min for 1 h through a sterile 80 mm diameter gelatin filter (Sartorius, Göttingen, Germany) with MD8 AirScan sampling device (Sartorius) placed on chair. Samples were collected approximately 1.0 m above ground level. Sampling was performed twice a month in each room before planting (Jan, Feb) and after planting (May, Jun, Jul, Aug, Sep). However, Sampling in January was only conducted once a month. Duplicate samples were collected at the same time, for a total of 78 samples. The gelatin filters after sampling were stored at 4 °C prior to DNA extraction. Natural materials were sampled at one location close to the sampling point of airborne microbiome in each room (Supplementary Fig. S1).

### DNA extraction

After the gelatin filters were dissolved in 50 mL of water treated with diethyl pyrocarbonate (DEPC) (Nalgene, Rochester, NY) at 50 °C, the dissolved solution was passed through 0.2 μm of analysis-filter (Thermo Fisher Scientific, Waltham, MA). Total DNA was extracted by the PowerWater DNA isolation kit (QIAGEN, Hilden, Germany) following the manufacture’s protocol. The final volume of isolated DNA was 100 μL in elution buffer (Tris–HCl, pH 8.0) included with the kit, and samples were stored at –20 °C for further use. To evaluate potential contamination, DNA extraction from blank filters, which are unused gelatin filters, was also performed using the same protocol as for the air samples. To extract total DNA from soil and wood-chip samples, the PowerSoil DNA isolation kit (QIAGEN) was used. To extract total DNA from the surface of plant leaves and the water in each room, the PowerWater DNA isolation kit was used after wiping the plant surface with swab soaked with swab solution (1% (v/v) Triton X-100, 0.5% (v/v) Tween 20, Tris–EDTA, pH8.0).

### Quantitative PCR (qPCR)

DNA extracted from samples was subjected to qPCR analysis using the ABI7500 system (Applied Biosystems, Bedford, MA) with forward primer 27F 5′-AGRGTTTGATYMTGGCTCAG-3′, and reverse primer 338R 5′-TGCTGCCTCCCGTAGGAGT-3′. qPCR was performed in 25 μL reaction volumes containing 10 μL template DNA, 12.5 μL of 2 × GeneAce SYBR qPCR mix alpha Low ROC (Nippon Gene, Tokyo, Japan), 0.5 μL of each primer (10 μM), and 1.5 μL of DEPC-treated water (Nalgene). qPCR was held at 95 °C for 10 min, followed by 45 cycles of 95 °C for 30 s, 60 °C for 1 min, and 72 °C for 30 s, and then a final extension at 72 °C for 5 min. A ZymoBIOMICS Microbial community DNA standard (Zymo Research, Irvine, CA) was used a qPCR standard. Serial dilution of the DNA standard was used to generate standard curves for qPCR. Duplicate aliquots of the standards and samples were included in each PCR run.

### 16S rRNA gene amplification followed by Illumina iSeq

For each sample, the V1–V2 region of the 16S rRNA gene was amplified using the primer set 27Fmod (5′-TCGTCGGCAGCGTCAGATGTGTATAAGAGACAGAGRGTTTGATYMTGGCTCAG-3′) and 338R (5′-GTCTCGTGGGCTCGGAGATGTGTATAAGAGACAGTGCTGCCTCCCGTAGGAGT-3′). The 16S rRNA gene was amplified in 25 μL reaction volumes containing 2.5 μL template DNA, 12.5 μL of 2 × KAPA HiFi HotStart ReadyMix (KAPA Biosystems, Wilmington, MA), and 5 μL of each primer (1.0 μM). Reactions were held at 95 °C for 3 min, followed by 25 cycles of 95 °C for 30 s, 55 °C for 30 s, and 72 °C for 30 s, and then a final extension at 72 °C for 5 min. Amplified PCR products were purified using the AMPure XP system (Beckman Coulter, Brea, CA) and eluted in 52.5 μL Tris–HCL in DEPC-treated water. Then, 2.5 μL of the purified PCR product was used as a template for the second-round PCR reaction to attach Nextera XT indices and sequencing adapters (Illumina, San Diego, CA). PCR was performed at 95 °C for 3 min, followed by eight cycles of 95 °C for 30 s, 55 °C for 30 s, and 72 °C for 30 s, and then a final extension at 72 °C for 5 min. DNA sequencing was performed with the iSeq 100 system (Illumina) by 151-bp paired-end reads.

### Bioinformatics and statistical analysis

Demultiplexed forward FASTQ files were used as the input file of the bioinformatics analysis. Each sample read was pre-processed and quality filtered and analyzed using QIIME2, version 2018.11^43^. Initially, the forward primer sequence (5′-AGRGTTTGATYMTGGCTCAG-3′) was trimmed by cutadapt1.18 command from demultiplexed FASTQ files^44^. The trimmed sequences were denoised with DADA2 software package^45^. For taxonomic classification, we performed the q2-feature-classifier which was trained based upon the SILVA database ver138^46,47^. In addition, we used RESCRIPt, a QIIME2 plug-in, to conduct taxonomy assignment only in the V1 and V2 regions^48^. Sequences matching cyanobacteria (which included the chloroplast) and mitochondria were removed, and sequences that identified phylum were used in the analysis. Both alignment mafft^49^ and the alignment mask commands were used to generate multiple aligned sequences. Both phylogeny fasttree^50^ and phylogeny midpoint-root commands were used to produce the phylogenic tree. Rarefaction curve analysis of the data was used to estimate the completeness of microbial community sampling. Several alpha metrics (observed ASVs, Shannon’s diversity index) were computed, and several beta diversity metrics (Jaccard distance, Bray–Curtis dissimilarity) were plotted by Emperor^51^. Group significance between alpha and beta diversity indexes was calculated with the QIIME2 plug-in using the Kruskal–Wallis pairwise test and permutational multivariate analysis of variance (PERMANOVA), respectively. Source-tracker is a Bayesian approach program to estimate the proportion of exogenous sequences in a given community that come from possible source environments^27^. The latest version of SourceTracker2 was used for source estimation of bioaerosol samples.

### Complies with international, national and/or institutional guidelines

The experimental research on plants in the present study complies with relevant institutional, national, and international guidelines and legislation.

## Supplementary Information

Supplementary Information.

## Data Availability

All sequencing data from this study were deposited in DNA Data Bank of Japan (DDBJ) under BioProject accession number PRJDB14671.
