# Supplementary material for: Diversity and compositional differences of the airborne microbiome in a biophilic indoor environment
Source: Sci Rep. 2023 May 20;13:8179. doi: 10.1038/s41598-023-34928-9 (PMC10199911; doi:10.1038/s41598-023-34928-9)
Supplement: Supplementary file 1 — Supplementary Information. [file 41598_2023_34928_MOESM1_ESM.docx]

**Diversity and compositional differences of the airborne microbiome in a biophilic indoor environment**

Akinobu Toyoda^1^, Yusuke Shibata^1^, Yuzy Matsuo^1^, Kumi Terada^1^, Hiroki Sugimoto^2^, Koichi Higashi^3^, Hiroshi Mori^3^, Akinori Ikeuchi^1^, Masakazu Ito^1^, Ken Kurokawa^3^、Satoshi Katahira^1,*^

^1^Frontier Research Center, Toyota Motor Corporation, Toyota, Aichi, 471-8572, Japan

^2^Toyota Central R&D Labs, Inc., Nagakute, Aichi 480-1192, Japan

^3^Department of Informatics, National Institute of Genetics, Mishima, Shizuoka, 411-8540, Japan

^*^e1355@mosk.tytlabs.co.jp

## **Supplementary Figure S1.** Layout of natural materials in the planting rooms.

## **Supplementary Figure S2.** Sequencing depth and rarefaction curve of the samples.

## **Supplementary Figure S3.** The number of relative reads in representative taxa sampled from natural materials in each room.

## **Supplementary Figure S4.** The number of airborne particles in the planting rooms.

## **Supplementary Figure S5.** Source-tracker analysis of the empty rooms.

## **Supplementary Table S1.** Natural materials in the planting rooms.

## **Supplementary Table S2.** Time-series variation in the diversity of the airborne microbiome at ASV level.

## **Supplementary Table S3.** Results of PERMANOVA in PCoA.

**Supplementary Table S4.** Scientific names and average leaf surface area of plants introduced into the planting rooms.


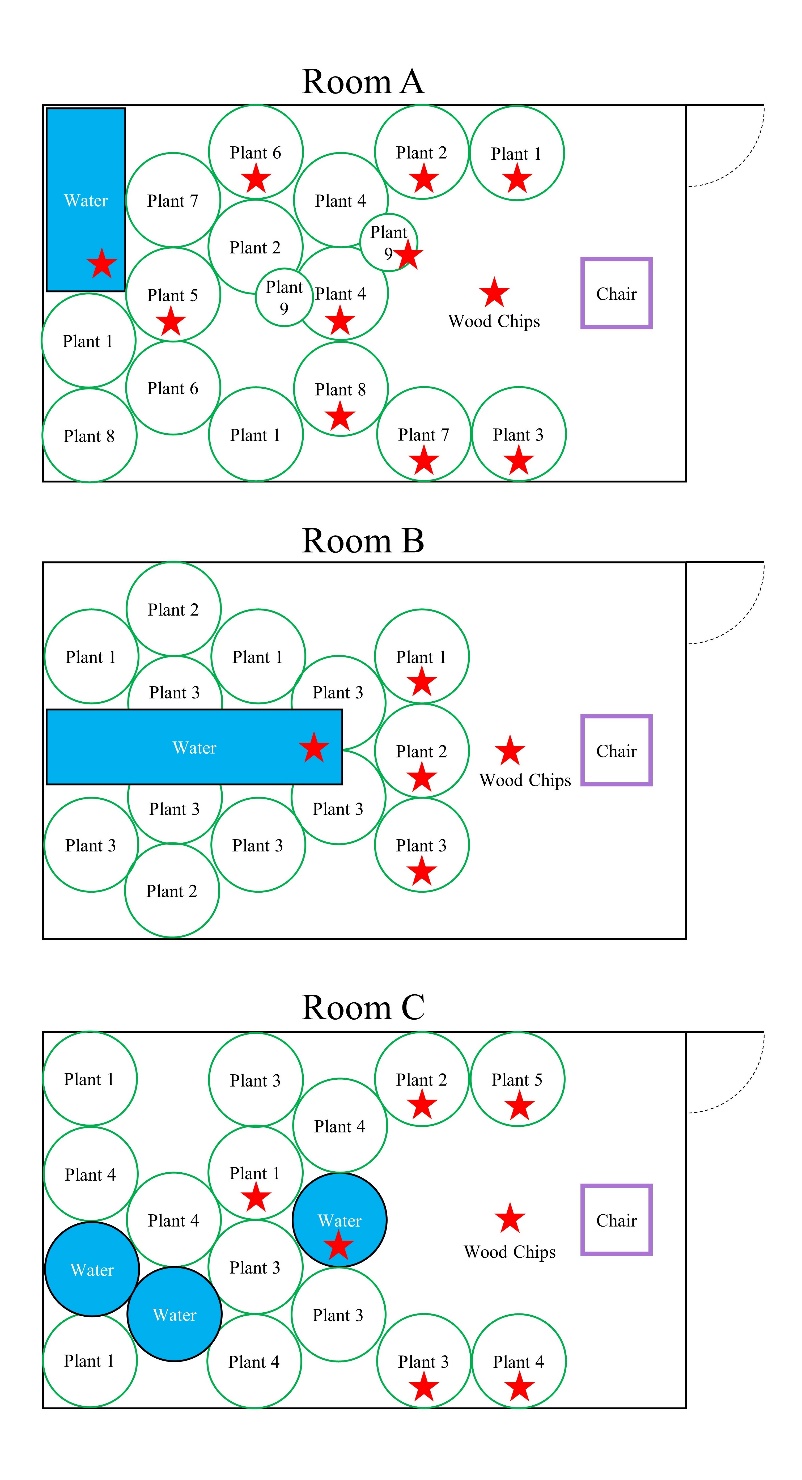


## **Supplementary Figure S1.** Layout of natural materials in the planting rooms.

The location of natural materials and sampling points (star mark) in each planting room (Rooms A–C) are shown. Wood chips cover the soil of the plant pods and floor. The pods of “Plant 9” in Room A hang from the ceiling.


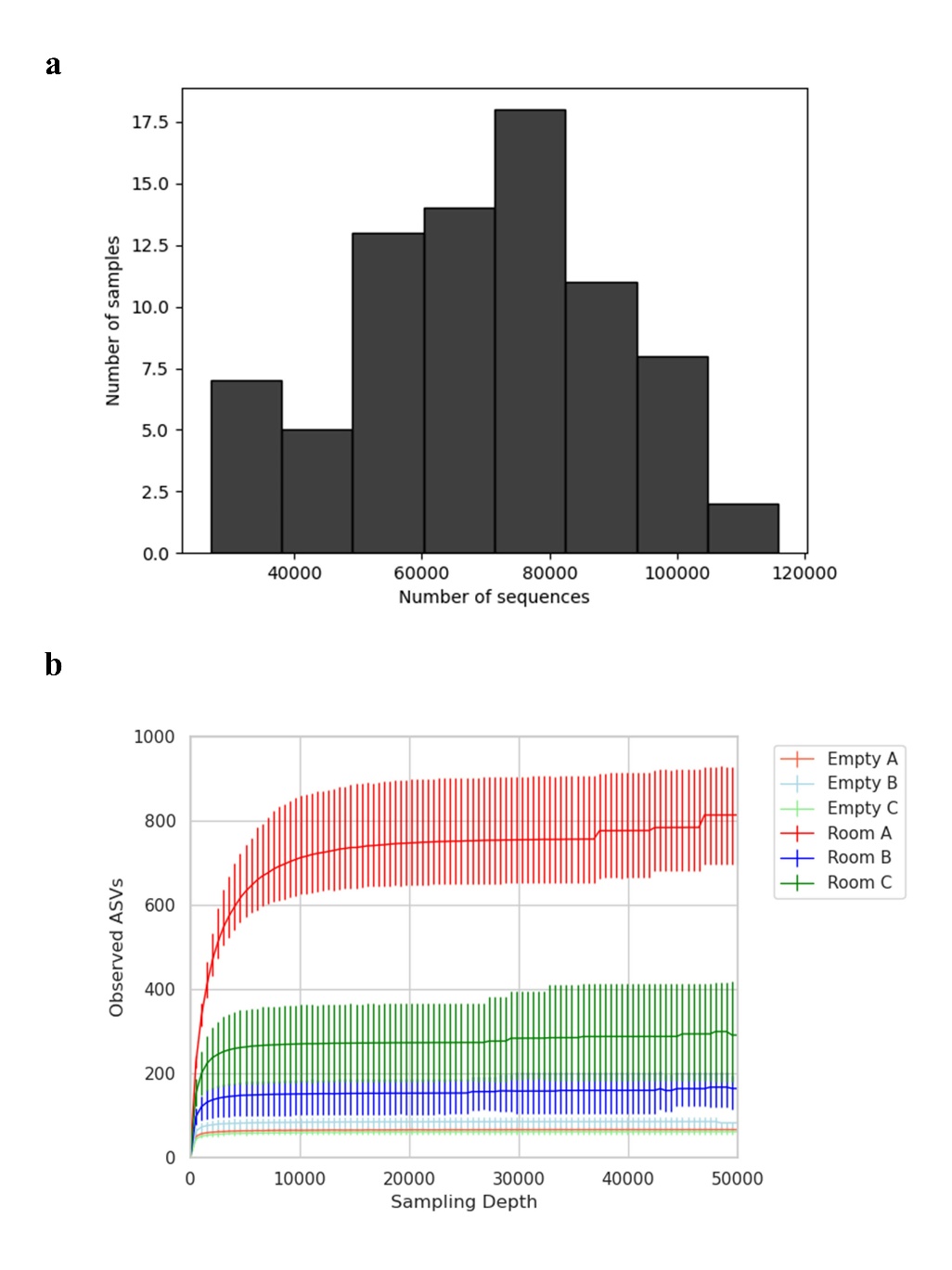


## **Supplementary Figure S2.** Sequencing depth and rarefaction curve of the samples.

(**a**) The sequencing depth histogram represents the number of samples as a function of the number of reads. (**b**) The rarefaction curves represent the average of observed ASVs as a function of the number of reads in each sampling room. The error bars of curves represent 25–75% quantiles.


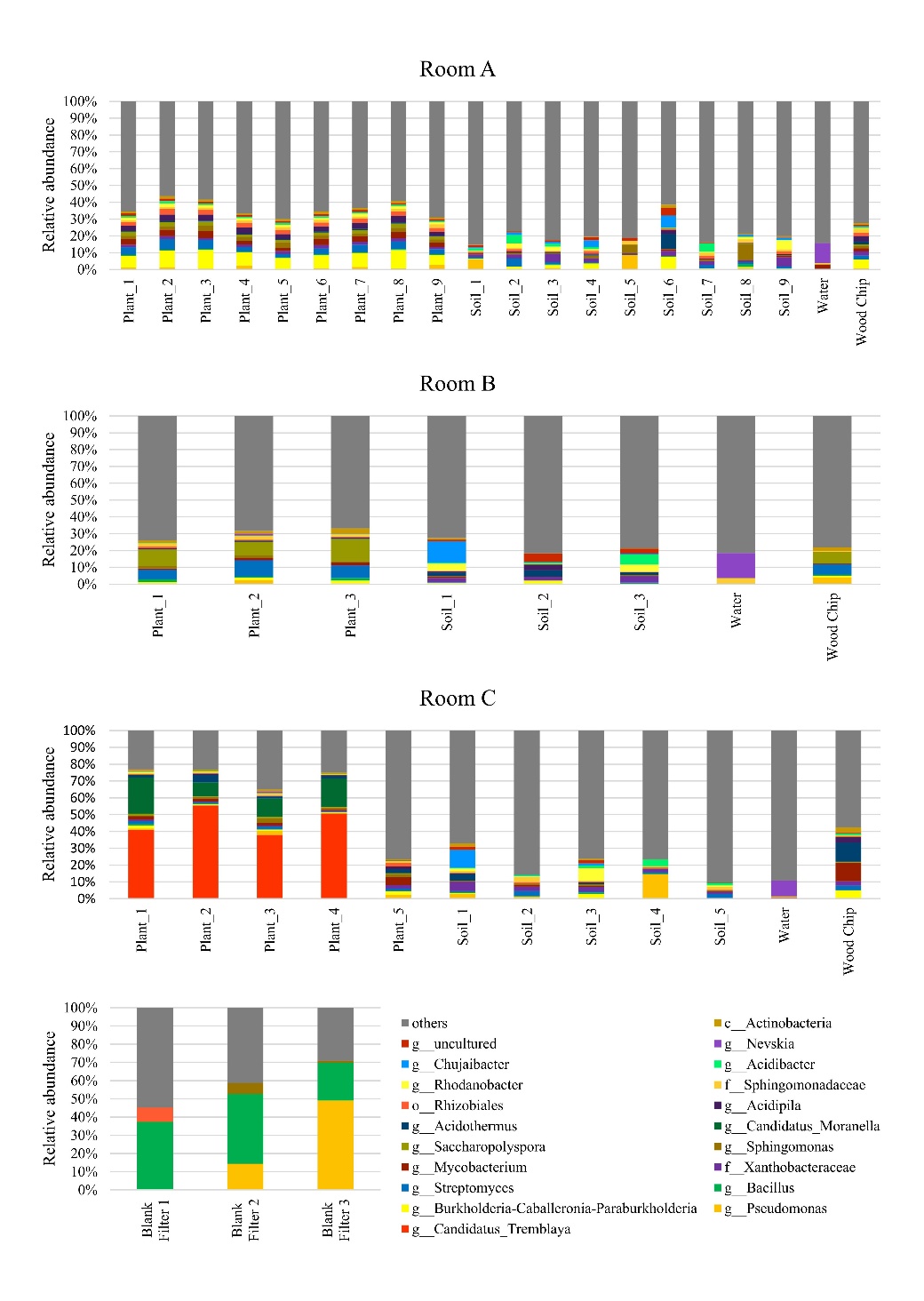


## **Supplementary Figure S3.** The number of relative reads in representative taxa sampled from natural materials in each room.

Bar plots show the most abundant bacterial taxa in each of the natural materials from planting rooms (Rooms A–C) at the genus level or higher taxonomic level.

**
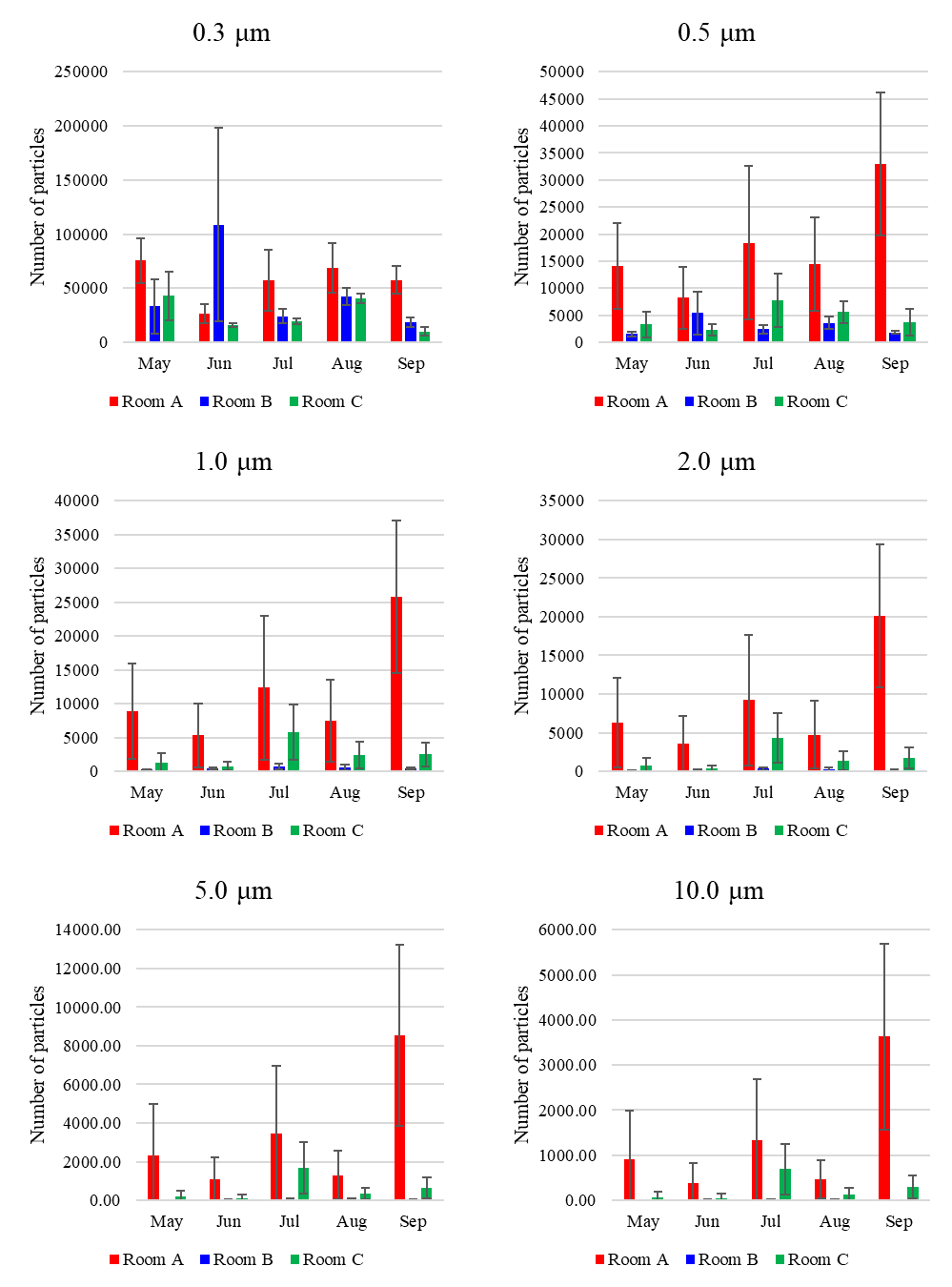
**

**Supplementary Figure S4.** The number of airborne particles in the planting rooms.

The bar graph shows the monthly average number of particles in 14.16 L of air in each planting room measured with the MET ONE HHPC6+ handheld particle counter (Beckman Coulter, Inc., Brea, CA), and the error bars represent the standard deviation.


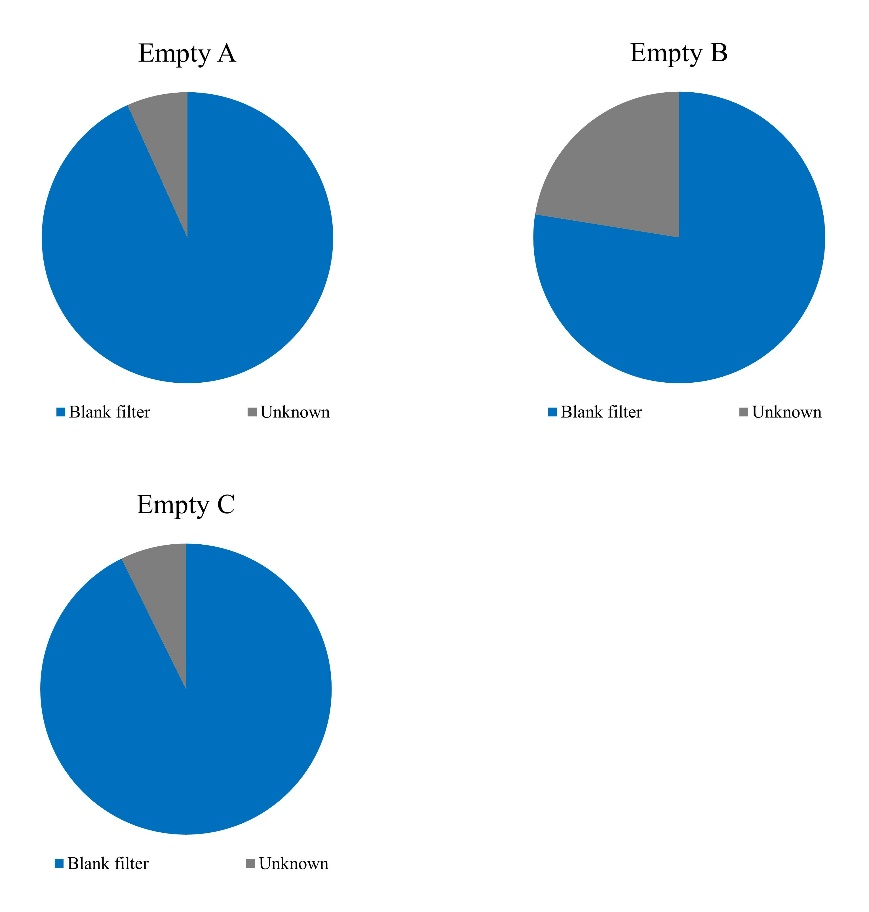


## **Supplementary Figure S5.** Source-tracker analysis of the empty rooms.

Pie charts show the proportion of blank filters in the airborne microbiome of the empty rooms.

## **Supplementary Table S1.** Natural materials in the planting rooms.

| Room | Type | Label | Details |
| --- | --- | --- | --- |
| Room A | Plant A | Plant 1 | *Spathiphyllum Schott* |
|  |  | Plant 2 | *Cissus rhombifolia* |
|  |  | Plant 3 | *Ficus benghalensis* |
|  |  | Plant 4 | *Anthurium hookeri* |
|  |  | Plant 5 | *Monstera deliciosa* |
|  |  | Plant 6 | *Goeppertia makoyana* |
|  |  | Plant 7 | *Leptochilus pteropus* |
|  |  | Plant 8 | *Strelitzia nicolai* |
|  |  | Plant 9 | *Scindapsus pictus* |
|  | Soil A | Soil 1-9 | obtained from each plant pot |
|  | Wood chip A | Wood chip | Wood chips of cedar and cypress bark |
|  | Water A | Water | Artificial waterfall |
| Room B | Plant B | Plant 1 | *Rhapis humilis* |
|  |  | Plant 2 | *Davallia tyermannii* |
|  |  | Plant 3 | *Liriope muscari* |
|  | Soil B | Soil 1-3 | obtained from each plant pot |
|  | Wood chip B | Wood chip | Wood chips of cedar and cypress |
|  | Water B | Water | Water channels |
| Room C | Plant C | Plant 1 | *Asparagus plumosus var. nanus* |
|  |  | Plant 2 | *Schefflera arboricola* |
|  |  | Plant 3 | *Nephrolepis exaltata ‘Teddy Junior’* |
|  |  | Plant 4 | *Albizia splendens* |
|  |  | Plant 5 | *Pteris cretica 'Albolineata'* |
|  | Soil C | Soil 1-5 | obtained from each plant pot |
|  | Wood chip C | Wood chip | Wood chips of pine bark |
|  | Water C | Water | Water bansins |

## **Supplementary Table S2.** Time-series variation in the diversity of the airborne microbiome at ASV level.

|  | Empty | | After planting | | | | |
| --- | --- | --- | --- | --- | --- | --- | --- |
|  | Jan | Feb | May | Jun | Jul | Aug | Sep |
| Number of ASVs observed in all three experimental rooms | 32 | 45 | 91 | 55 | 93 | 86 | 76 |
| Total number of ASVs observed in three experimental rooms | 284 | 426 | 2291 | 2545 | 2672 | 3326 | 2998 |
| Percentage of ASVs observed in all three experimental rooms | 11.3% | 10.6% | 4.0% | 2.2% | 3.5% | 2.6% | 2.5% |

## **Supplementary Table S3.** Results of PERMANOVA in PCoA.

**a**

| Group 1 | Group 2 | Sample size | Permutations | pseudo-F | p-value | q-value |
| --- | --- | --- | --- | --- | --- | --- |
| Empty A | Empty B | 12 | 999 | 1.116704 | 0.067 | 0.077308 |
| Empty A | Empty C | 12 | 999 | 0.928848 | 0.834 | 0.834 |
| Empty A | Room A | 26 | 999 | 6.448328 | 0.001 | 0.00125 |
| Empty A | Room B | 26 | 999 | 3.742143 | 0.001 | 0.00125 |
| Empty A | Room C | 26 | 999 | 3.892591 | 0.001 | 0.00125 |
| Empty B | Empty C | 12 | 999 | 1.066263 | 0.2 | 0.214286 |
| Empty B | Room A | 26 | 999 | 5.506263 | 0.001 | 0.00125 |
| Empty B | Room B | 26 | 999 | 3.197686 | 0.001 | 0.00125 |
| Empty B | Room C | 26 | 999 | 3.254582 | 0.001 | 0.00125 |
| Empty C | Room A | 26 | 999 | 6.559335 | 0.001 | 0.00125 |
| Empty C | Room B | 26 | 999 | 3.796286 | 0.001 | 0.00125 |
| Empty C | Room C | 26 | 999 | 3.984177 | 0.001 | 0.00125 |
| Room A | Room B | 40 | 999 | 9.115027 | 0.001 | 0.00125 |
| Room A | Room C | 40 | 999 | 6.711314 | 0.001 | 0.00125 |
| Room B | Room C | 40 | 999 | 5.047654 | 0.001 | 0.00125 |

**b**

| Group 1 | Group 2 | Sample size | Permutations | pseudo-F | p-value | q-value |
| --- | --- | --- | --- | --- | --- | --- |
| Empty A | Empty B | 12 | 999 | 1.484409 | 0.076 | 0.084643 |
| Empty A | Empty C | 12 | 999 | 1.343583 | 0.079 | 0.084643 |
| Empty A | Room A | 26 | 999 | 37.90209 | 0.001 | 0.00125 |
| Empty A | Room B | 26 | 999 | 11.53728 | 0.001 | 0.00125 |
| Empty A | Room C | 26 | 999 | 9.747694 | 0.001 | 0.00125 |
| Empty B | Empty C | 12 | 999 | 1.500431 | 0.089 | 0.089 |
| Empty B | Room A | 26 | 999 | 31.20496 | 0.001 | 0.00125 |
| Empty B | Room B | 26 | 999 | 11.58093 | 0.001 | 0.00125 |
| Empty B | Room C | 26 | 999 | 9.385406 | 0.001 | 0.00125 |
| Empty C | Room A | 26 | 999 | 38.43004 | 0.001 | 0.00125 |
| Empty C | Room B | 26 | 999 | 13.0151 | 0.001 | 0.00125 |
| Empty C | Room C | 26 | 999 | 10.78069 | 0.001 | 0.00125 |
| Room A | Room B | 40 | 999 | 50.33119 | 0.001 | 0.00125 |
| Room A | Room C | 40 | 999 | 38.41437 | 0.001 | 0.00125 |
| Room B | Room C | 40 | 999 | 16.15311 | 0.001 | 0.00125 |

Tables show results of the statistical analysis using PERMANOVA at (**a**) the Jaccard distance and (**b**) Bray-Curtis dissimilarity for empty and planting rooms.

## **Supplementary Table S4.** Scientific names and average leaf surface area of plants introduced into the planting rooms.

| Average area per leaf (cm^2^) | 164.46 | 33.31 | 275.00 | 814.53 | 2,743.60 | 320.22 | 221.75 | 4,551.61 | 53.15 | 93.50 | 204.08 | 105.14 | 243.11 | 29.02 | 308.08 | 75.71 | 63.11 |
| --- | --- | --- | --- | --- | --- | --- | --- | --- | --- | --- | --- | --- | --- | --- | --- | --- | --- |
| Species | *Spathiphyllum Schott* | *Cissus rhombifolia* | *Ficus benghalensis* | *Anthurium hookeri* | *Monstera deliciosa* | *Goeppertia makoyana* | *Leptochilus pteropus* | *Strelitzia nicolai* | *Scindapsus pictus* | *Rhapis humilis* | *Davallia tyermannii* | *Liriope muscari* | *Asparagus plumosus var. nanus* | *Schefflera arboricola* | *Nephrolepis exaltata ‘Teddy Junior’* | *Albizia splendens* | *Pteris cretica 'Albolineata'* |
| Genus | *Spathiphyllum* | *Cissus* | *Ficus* | *Anthurium* | *Monstera* | *Goeppertia* | *Leptochilus* | *Strelitzia* | *Scindapsus* | *Rhapis* | *Liriope* | *Asparagus* | *Schefflera* | *Schefflera* | *Nephrolepis* | *Albizia* | *Pteris* |
| Family | *Araceae* | *Vitaceae* | *Moraceae* | *Araceae* | *Araceae* | *Marantaceae* | *Polypodiaceae* | *Strelitziaceae* | *Araceae* | *Araceae* | *Davalliaceae* | *Asparagaceae* | *Asparagaceae* | *Araliaceae* | *Nephrolepidaceae* | *Fabales* | *Polypodiales* |
| Order | *Alismatales* | *Vitales* | *Rosales* | *Rosales* | *Rosales* | *Zingiberales* | *Polypodiales* | *Zingiberales* | *Alismatales* | *Alismatales* | *Polypodiales* | *Asparagales* | *Asparagales* | *Apiales* | *Polypodiales* | *Fabales* | *Polypodiales* |
| Class | *Liliopsida* | *Magnoliopsida* | *Magnoliopsida* | *Liliopsida* | *Liliopsida* | *Liliopsida* | *Polypodiopsida* | *Liliopsida* | *Liliopsida* | *Liliopsida* | *Polypodiopsida* | *Liliopsida* | *Liliopsida* | *Magnoliopsida* | *Polypodiopsida* | *Magnoliopsida* | *Polypodiopsida* |
| Label | Plant 1 | Plant 2 | Plant 3 | Plant 4 | Plant 5 | Plant 6 | Plant 7 | Plant 8 | Plant 9 | Plant 1 | Plant 2 | Plant 3 | Plant 1 | Plant 2 | Plant 3 | Plant 4 | Plant 5 |
| Plant | Plant A |  |  |  |  |  |  |  |  | Plant B |  |  | Plant C |  |  |  |  |
